# LncRNA‐RMRP promotes nucleus pulposus cell proliferation through regulating miR‐206 expression

**DOI:** 10.1111/jcmm.13817

**Published:** 2018-08-29

**Authors:** Xuesong Wang, Lei Peng, Xiaojin Gong, Xiugong Zhang, Ruifu Sun, Jinlong Du

**Affiliations:** ^1^ Spinal Department of Qingdao Central Hospital Qingdao Central Hospital Qingdao China; ^2^ Library of QingDao Central Hospital Qingdao Central Hospital Qingdao China

**Keywords:** intervertebral disc degeneration, lncRNAs, miR‐206, RMRP

## Abstract

Long noncoding RNAs (LncRNAs) are involved in the pathogenesis of intervertebral disc degeneration (IDD). However, the biological function and expression of RMRP were still unclear. In our study, we showed that RMRP expression was up‐regulated in degenerated NP tissues compared to normal NP samples, and higher RMRP expression was associated with the disc degeneration grade. Further studies indicated that ectopic expression of RMRP enhanced NP cell growth and also enhanced the expression of ki‐67, PCNA and cyclin D1 in the NP cell. Moreover, overexpression of RMRP promoted the expression of Type II collagen and aggrecan and suppressed the expression of MMP13 and ADAMTS4. In addition, we found that the expression of miR‐206 was down‐regulated in degenerated NP tissues compared to normal NP samples, and lower miR‐206 expression was correlated with the disc degeneration grade. Interestingly, we indicated that miR‐206 expression in NP tissues was negatively correlated with the expression of RMRP. Ectopic expression of miR‐206 suppressed NP cell proliferation and suppressed the expression of Type II collagen and aggrecan and enhanced the expression of MMP13 and ADAMTS4. Furthermore, we demonstrated that overexpression of RMRP increased NP cell growth and regulated ECM expression through targeting miR‐206. These results suggested that lncRNA‐RMRP promoted the progression of IDD through targeting miR‐206, providing an attractive new therapeutic approach for the treatment of IDD disease.

## 1 INTRODUCTION

Chronic low back pain (LBP) influenced more than 70% people at some time in their lives, with about 10% people becoming chronic disabled.^1^, ^2^, ^3^ The aetiology of LBP is considered to be multifactorial, and intervertebral disc degenerative (IDD) contributes to the development of LBP.^3^, ^4^, ^5^ The cause of IDD is thought to associate with many aetiological factors such as ageing, lifestyle and genetic predisposition.^6^, ^7^, ^8^, ^9^ Recent research in cell molecular biology may contemplate treating intervertebral disc itself at the molecular biology level to delay or prevent the development of IDD.^10^, ^11^, ^12^, ^13^, ^14^, ^15^ However, the underlying molecular and cellular mechanism of IDD progression is still unknown. Thus, it is important for us to study the mechanism of IDD and to find new treatment strategies for IDD.

Long noncoding RNAs (LncRNAs) are longer than 200 nucleotides (nts) in length with no or limited protein‐coding capacity and were found to play important roles in diverse human diseases and biological processes.^16^, ^17^, ^18^, ^19^, ^20^, ^21^ Mounting evidence has suggested that many lncRNAs are beregulated in diverse diseases such as tumours, diabetes, coronary heart disease, osteoarthritis and also IDD.^22^, ^23^, ^24^, ^25^, ^26^, ^27^, ^28^, ^29^ For example, Lan and colleagues^30^ profiled lncRNA expression in degenerate disc tissues and normal adults' lumbar discs by microarray and found a total of 3081 differentially expressed lncRNAs. Wang et al^31^ showed that knockdown of RP11‐296A18.3 suppressed human NP cell proliferation and decreased the expression of collagen I and matrix metalloproteinase (MMP)‐13. Tan et al^32^ studied the role of SNHG1 in the pathogenesis of IDD. They indicated that the expression of SNHG1 was up‐regulated in the IDD samples compared to the control samples. Recently, a novel lncRNA‐RMRP was found to be deregulated in several diseases.^33^, ^34^, ^35^ For example, Meng et al^35^ demonstrated that the expression of RMRP was up‐regulated in lung cancer tissues and overexpression of RMRP enhanced lung cancer cell growth, invasion and colony formation through regulating miR‐206 expression. Moreover, Shao et al^36^ found that the RMRP expression was deregulated in gastric tumour. In addition, Feng et al^33^ showed that RMRP expression was up‐regulated in glioma samples compared to normal brain samples. Down‐regulation of RMRP inhibited glioma cell growth, invasion and migration. However, the biological function and expression of RMRP are still unknown.

In this study, we first measured the expression of RMRP in the intervertebral disc tissues and we found that RMRP expression was up‐regulated in degenerated NP tissues compared to normal NP samples, and higher RMRP expression was associated with the disc degeneration grade.

## 2 MATERIALS AND METHODS

### 2.1 Tissues

Degenerated IVD tissues were collected from IDD patients undergoing discectomy surgery. Nondegenerated IVD tissues were obtained from cases with scoliosis undergoing surgery. Routine MRI of spine was performed for all these cases before operation and the modified Pfirrmann classification was used to determine the degree of disc degeneration according to T2‐weighted images. Sample was washed three times in the physiological saline and IVD was separated using stereotaxic microscope and then the tissue was immediately frozen in liquid nitrogen. Informed consent was obtained from each patient and this study was approved by the ethics committee of QingDao Central Hospital.

### 2.2 RNA isolation and qRT‐PCR

Total RNA from samples specimens or cells was extracted with TRIzol reagent (Invitrogen, Carlsbad, CA, USA). Reverse‐transcribed was performed with cDNA Synthesis Kit (GeneCopoeia Inc., Rockville, MD, USA) following the manufacturer's information. Quantitative real‐time reverse transcription‐PCR (qRT‐PCR) was carried out to determine the expression level of miRNA and LncRNA expression using SYBR‐green PCR kit (GenePharma, Shanghai, China) on PRISM 7900HT system. GAPDH or U6 snRNA was performed to as normalization control and the expression was determined with 2‐DDCT method. qRT‐PCR primers were listed as following: RMRP Forward: 5′‐ACTCCAAAGTCCGCCAAGA‐3′′ and 5′‐GTAACTAGA−GGGAGCTGAC‐3′; GADPH: Forward: 5′‐GTCAA‐CGGATTTGGTCTGTATT‐3′′ and 5′‐AGTCTTCTGGGTGGCAGTGAT‐3′.

### 2.3 Cell cultures and cell transfection

Human NP cell was isolated from human NP tissues following to previous standardized method. NP samples were washed in PBS for three times and NP was separated from IVD samples using stereotaxic microscope and then cut to pieces. Then, these tissues were incubated in Type II collagenase in DMEM medium. After isolation, cells were cultured in the DMEM medium with penicillin, streptomycin and 10% FBS. pcDNA‐RMRP and pcDNA‐control vector were purchased from the Biosune biotechnology (Shanghai, China) and then transfected to the cell using DharmaFECT1 kit (Dharmacon, TX, USA) following to instruction.

### 2.4 Cell proliferation and cell cycle assays

Cell growth of NP cell was determined using the 3‐(4,5‐dimethylthiazol‐2‐yl)‐2,5‐diphenyltetrazolium bromide (MTT) method. Cells were cultured in 96‐well plate and MTT medium was added to each well. The absorbance was detected at the 490 nm using enzyme‐labelled analyser. For the cell cycle, cells were washed twice in PBS and fixed with ethanol overnight at 4°C. DNA content staining was performed with propidium iodide and ribonuclease A for a half hour. Population in G2–M, S and G0‐G1 phase was determined by flow cytometry (Beckman Coulter, Indianapolis, USA).

### 2.5 Western blot assay

Total protein from corresponding cell or samples was extracted by RIPA buffer (Pierce) with Protease Inhibitor (Pierce, Waltham, MA, USA). The concentration of protein was determined with BCA Protein Kit. Equivalent protein was electrophoresed by 12% SDS‐PAGE and transferred to the PVDF membranes. The membrane was blocked with nonfat milk and then was immunoblotted with primary antibodies (anti‐HDAC6 and anti‐GAPDH, Abcam, Cambridge, UK). After washed for three times in PBS, the membrane was immunoblotted with HRP‐linked secondary antibodies (Cell Signaling, Boston, MA, USA). The signal was determined by ECL detection system (Millipore).

### 2.6 Statistical analysis

Data were measured by SPSS Data (version 18, Inc., Chicago, IL, USA) and were represented as mean ± SD. The comparison among multiple groups was determined with one‐way ANOVA and the difference between two groups was measured with Student's *t* test. *P* < 0.05 was considered to be statistically significant.

## 3 RESULTS

### 3.1 The expression of RMRP was up‐regulated in degenerated NP tissues

**Figure 1:**
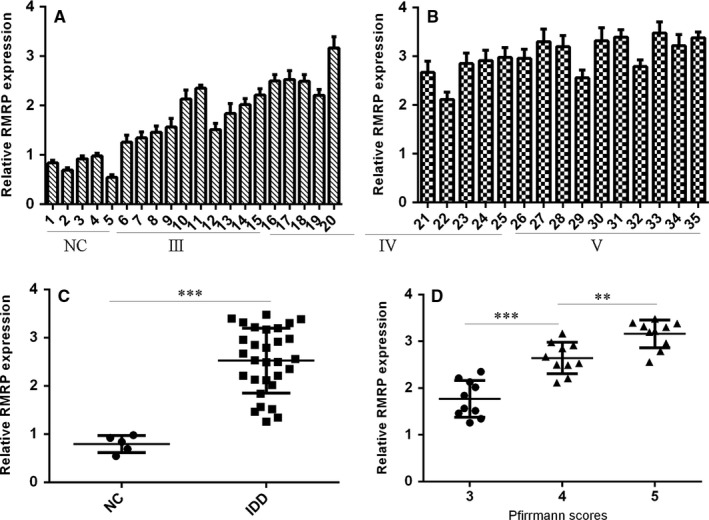
The expression of RMRP was up‐regulated in the degenerated NP tissues. A,B, The RMRP expression in the 5 normal NP tissues and 30 degenerated NP tissues was measured by qRT‐PCR. U6 was used as the internal control. C, The expression of RMRP was up‐regulated in degenerated NP tissues compared to normal NP samples. D, RMRP expression was higher with the disc degeneration grade. **P < 0.01 and ***P < 0.001

We first determined the expression level of RMRP in NP tissues. RMRP expression in the 5 normal NP tissues and 30 degenerated NP tissues was shown in Figure 1A,B. Furthermore, we indicated that the expression of RMRP was up‐regulated in degenerated NP tissues compared to normal NP samples (Figure 1C). Moreover, we showed that RMRP expression was higher in advanced the disc degeneration grade (Figure 1D).

### 3.2 The expression of miR‐206 was down‐regulated in degenerated NP tissues

**Figure 2:**
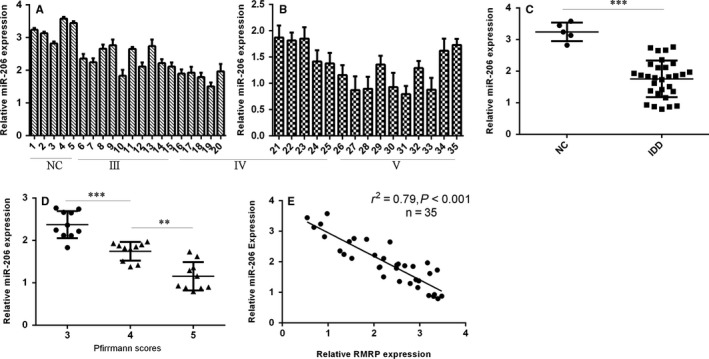
The expression of miR‐206 was down‐regulated in the degenerated NP tissues. A,B, The miR‐206 expression in the 5 normal NP tissues and 30 degenerated NP tissues was determined by qRT‐PCR assay. C, The expression of miR‐206 was down‐regulated in degenerated NP tissues compared to normal NP samples. D, miR‐206 expression was lower with the disc degeneration grade. E, miR‐206 expression in the NP tissues was negatively correlated with the expression of RMRP. **P < 0.01 and ***P < 0.001

Then, we determined the expression level of miR‐206 in NP tissues. miR‐206 expression in 5 normal NP tissues and 30 degenerated NP tissues was shown in Figure 2A,B. Furthermore, we indicated that the expression of miR‐206 was down‐regulated in degenerated NP tissues compared to normal NP samples (Figure 2C). Moreover, we showed that miR‐206 expression was lower in early disc degeneration grade (Figure 2D). In addition, we indicated that miR‐206 expression in NP tissues was negatively correlated with the expression of RMRP (Figure 2E).

### 3.3 Overexpression of RMRP suppressed the miR‐206 expression in the NP cell

**Figure 3:**
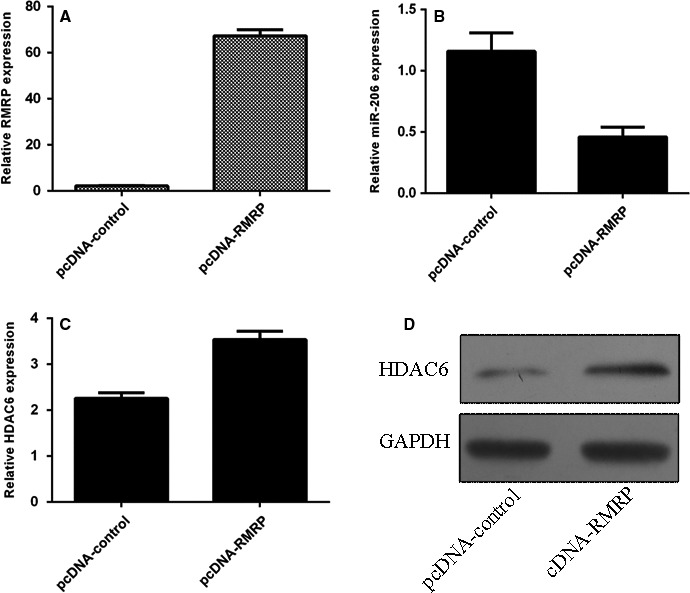
Overexpression of RMRP suppressed the miR‐206 expression in the NP cell. A, We confirmed that the expression of RMRP was significantly up‐regulated in the NP cell after transfected with pcDNA‐RMRP. B, Overexpression of RMRP suppressed the miR‐206 expression in the NP cell. C, Ectopic expression of RMRP promoted the HDAC6 expression in the NP cell. D, The protein expression of HDAC6 in the NP cell was detected by Western blot. GAPDH was used as the control

We confirmed that the expression of RMRP was significantly up‐regulated in NP cell after transfected with pcDNA‐RMRP (Figure 3A). Overexpression of RMRP suppressed miR‐206 expression in NP cell (Figure 3B). In addition, we showed ectopic expression of RMRP promoted HDAC6 expression in NP cell (Figure 3C). We also indicated that RMRP overexpression enhanced HDAC6 protein expression in NP cell (Figure 3D).

### 3.4 Ectopic expression of RMRP enhanced NP cell growth

**Figure 4:**
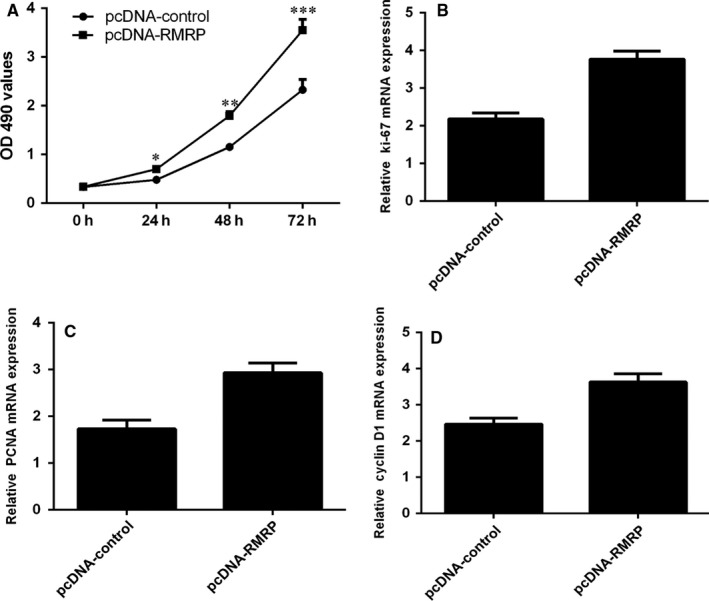
Ectopic expression of RMRP enhanced the NP cell growth. A, Ectopic expression RMRP promoted the NP cell proliferation. B, The expression of ki‐67 was measured by qRT‐PCR analysis. C, Overexpression of RMRP enhanced the PCNA expression in the NP cell. (D) Ectopic expression of RMRP promoted the expression of cyclin D1 in the NP cell. *P < 0.05, **P < 0.01 and ***P < 0.001

We next showed that ectopic expression RMRP promoted NP cell proliferation (Figure 4A). The expression of ki‐67 was up‐regulated in NP cell after transfected with pcDNA‐RMRP (Figure 4B). Overexpression of RMRP enhanced PCNA expression in NP cell (Figure 4C). Moreover, ectopic expression of RMRP promoted the expression of cyclin D1 in NP cell (Figure 4D).

### 3.5 RMRP overexpression increased Type II collagen and aggrecan expression and suppressed MMP13 and ADAMTS4 expression

**Figure 5:**
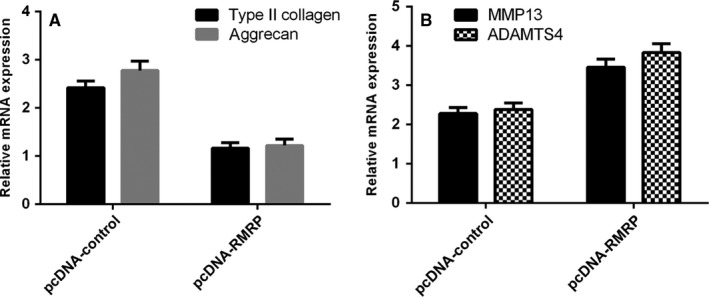
RMRP overexpression increased Type II collagen and aggrecan expression and suppressed MMP13 and ADAMTS4 expression. A, The Type II collagen and aggrecan expression was determined by qRT‐PCR assay. GAPDH was used as the control. B, Ectopic expression of RMRP decreased the expression of MMP13 and ADAMTS4 expression in the NP cell

We next indicated that overexpression of RMRP increased Type II collagen and aggrecan expression in the NP cell (Figure 5A). In addition, we showed that ectopic expression of RMRP decreased the expression of MMP13 and ADAMTS4 (Figure 5B).

### 3.6 Ectopic expression of miR‐206 suppressed NP cell proliferation and regulated the extracellular matrix (ECM) expression

**Figure 6:**
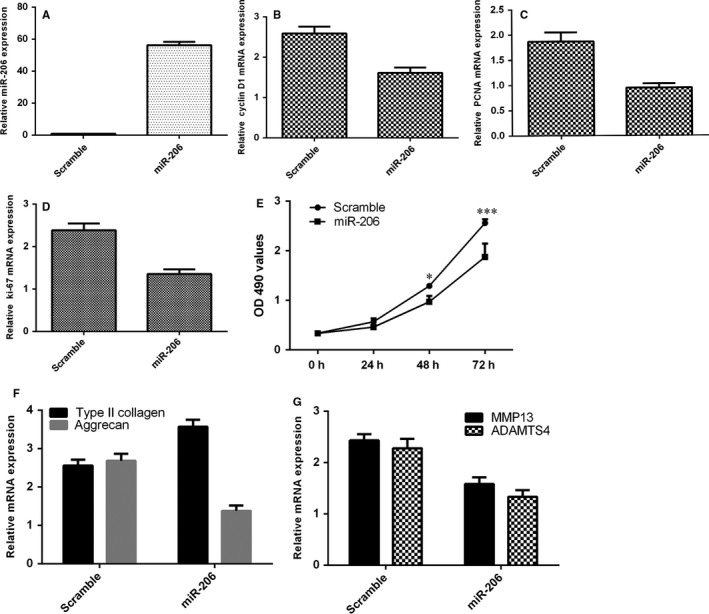
Ectopic expression of miR‐206 suppressed the NP cell proliferation and regulated the extracellular matrix (ECM) expression. A, The expression of miR‐206 was significantly up‐regulated in the NP cell after transfected with miR‐206 mimic. B, Overexpression of miR‐206 suppressed the NP cell proliferation. C, The ki‐67 expression was determined by qRT‐PCR assay. GAPDH was used as the control. D, The PCNA expression was measured by qRT‐PCR analysis. E, Ectopic expression of miR‐206 decreased the expression of cyclin D1 in the NP cell. F, Overexpression of miR‐206 suppressed the Type II collagen and aggrecan expression in the NP cell. G, Ectopic expression of miR‐206 promoted the expression of MMP13 and ADAMTS4 expression in the NP cell. *P < 0.05 and ***P < 0.001

The expression of miR‐206 was significantly up‐regulated in NP cell after transfected with miR‐206 mimic (Figure 6A). We found that overexpression of miR‐206 suppressed NP cell proliferation (Figure 6B). The expression of ki‐67 was down‐regulated in NP cell after transfected with miR‐206 mimic (Figure 6C). Overexpression of miR‐206 suppressed PCNA expression in NP cell (Figure 6D). Moreover, ectopic expression of miR‐206 decreased the expression of cyclin D1 in NP cell (Figure 6E). Furthermore, we showed that overexpression of miR‐206 suppressed Type II collagen and aggrecan expression in NP cell (Figure 6F). In addition, we showed that ectopic expression of miR‐206 promoted the expression of MMP13 and ADAMTS4 in NP cell (Figure 6G).

### 3.7 Ectopic expression of RMRP increased NP cell growth and regulated ECM expression through targeting miR‐206

**Figure 7:**
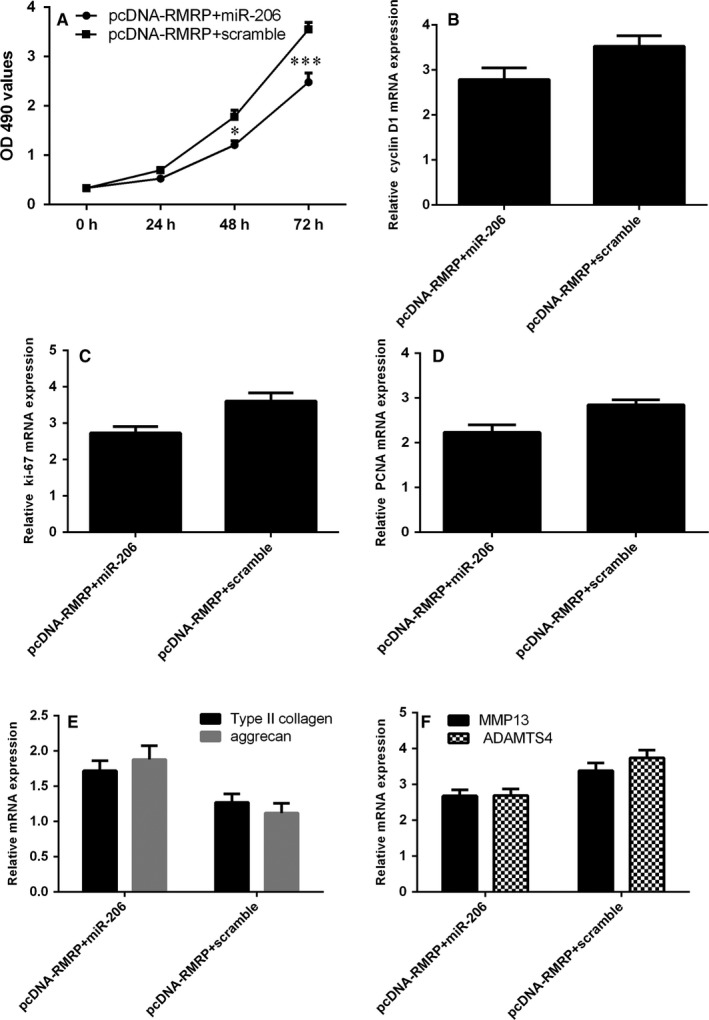
Ectopic expression of RMRP increased NP cell growth and regulated ECM expression through targeting miR‐206 expression. A, Cell proliferation was determined by MTT method. B, The cyclin D1 expression in the NP cell was detected by qRT‐PCR. C, The ki‐67 expression in the NP cell was detected by qRT‐PCR. D, The PCNA expression in the NP cell was detected by qRT‐PCR. E, Ectopic expression of RMRP increased Type II collagen and aggrecan expression in the pcDNA‐RMRP‐overexpressing NP cell. F, The expression of MMP13 and ADAMTS4 was measured by qRT‐PCR. *P < 0.05 and ***P < 0.001

To study the biological function association between RMRP and miR‐206, we employed loss and gain‐of‐function approaches. We showed that the cell proliferation was down‐regulated in the group of pcDNA‐RMRP and scramble compared to pcDNA‐RMRP and miR‐206 mimic group (Figure 7A). In addition, we showed that ectopic expression of miR‐206 suppressed the expression of cyclin D1 (Figure 7B), ki‐67(Figure 7C) and PCNA (Figure 7D) in the pcDNA‐RMRP‐overexpressing NP cell. Moreover, we indicated that ectopic expression of RMRP increased Type II collagen and aggrecan expression (Figure 7E) and suppressed MMP13 and ADAMTS4 (Figure 7F) expression through inhibiting miR‐206 expression.

## 4 DISCUSSION

In our study, we studied the expression and biological function of lncRNA‐RMRP in the degenerated intervertebral disc. We showed that RMRP expression was up‐regulated in degenerated NP tissues compared to normal NP samples and RMRP expression was associated with the disc degeneration grade. Further studies indicated that ectopic expression of RMRP enhanced NP cell growth and also enhanced the expression of ki‐67, PCNA and cyclin D1 in the NP cell. Moreover, overexpression of RMRP promoted Type II collagen and aggrecan expression and suppressed the expression of MMP13 and ADAMTS4. In addition, we found that the expression of miR‐206 was down‐regulated in degenerated NP tissues compared to normal NP samples and lower miR‐206 expression was correlated with early disc degeneration grade. Interestingly, we indicated that miR‐206 expression in NP tissues was negatively correlated with the expression of RMRP. Ectopic expression of miR‐206 suppressed NP cell proliferation and suppressed Type II collagen and aggrecan expression and enhanced the expression of MMP13 and ADAMTS4. Furthermore, we demonstrated that overexpression of RMRP increased NP cell growth and regulated ECM expression through targeting miR‐206.

A growing number of studies indicated that lncRNAs were correlated with the development of several diseases.^23^, ^37^, ^38^, ^39^ The role of lncRNAs in the development of IDD has also been explored.^29^, ^31^, ^40^ For example, Wang et al^41^ found that lncRNA linc‐ADAMTS5 cooperates with RREB1 to suppress ADAMTS5 expression, then affecting ECM degeneration of the IVD. Chen et al^42^ indicated that TUG1 expression was up‐regulated in degenerated IVD tissues and positively associated with β‐catenin and Wnt expression. Inhibited expression of TUG1 inhibited the expression of β‐catenin, Bax, ADAMTS5, MMP3, Caspase‐3 and Wnt1 and promoted the COL2A1, Aggrecan and Bcl‐2 expression. Xi et al^43^ demonstrated that the expression of HCG18 was up‐regulated in IDD patients and higher expression of HCG18 was correlated with the grade of disc degeneration. In addition, they showed that HCG18 inhibited NP cell growth and enhanced the development of IDD through the NFκB/TRAF6/miR‐146a‐5p axis. LncRNA‐RMRP is one new LncRNA that was deregulated in several diseases. For example, Shao et al^36^ found that RMRP expression was deregulated in gastric tumour. Moreover, Meng et al^35^ demonstrated that the expression of RMRP was up‐regulated in lung cancer tissues and overexpression of RMRP enhanced lung cancer cell growth, invasion and colony formation through regulating miR‐206 expression. In addition, Feng et al^33^ showed that RMRP expression was up‐regulated in glioma samples compared to normal brain samples. Down‐regulation of RMRP inhibited glioma cell growth, invasion and migration. However, the biological function and expression of RMRP were still unknown. In our study, we indicated that the expression of RMRP was up‐regulated in degenerated NP tissues compared to normal NP samples and higher RMRP expression was correlated with the disc degeneration grade. Further studies showed that overexpression of RMRP promoted NP cell proliferation and also increased the expression of ki‐67, PCNA and cyclin D1 in the NP cell. Moreover, ectopic expression of RMRP increased the Type II collagen and aggrecan expression and decreased the expression of MMP13 and ADAMTS4.

Recently, increasing evidence has suggested that lncRNAs function as competing endogenous RNAs (ceRNAs) to target miRNA expression.^44^, ^45^, ^46^ For examples, Shao et al^36^ showed that RMRP regulated gastric cell cycle and cyclin D2 expression through regulating miR‐206 expression. In line with this, we also showed that overexpression of RMRP suppressed miR‐206 expression in NP cell. Liu et al^47^ showed that miR‐206 suppressed head and neck squamous cell carcinoma cell growth, invasion and migration through targeting HDAC6 via mTOR/PTEN/AKT pathway. Pang et al^48^ indicated that miR‐206 overexpression suppressed hepatocellular carcinoma cell growth by regulating CDK9 expression. In our study, we indicated that the expression of miR‐206 was down‐regulated in degenerated NP tissues compared to normal NP samples, and lower miR‐206 expression was correlated with the disc degeneration grade. Interestingly, we indicated that miR‐206 expression in NP tissues was negatively correlated with the expression of RMRP. Ectopic expression of miR‐206 suppressed NP cell proliferation and suppressed the Type II collagen and aggrecan expression and enhanced the expression of MMP13 and ADAMTS4. Furthermore, we demonstrated that overexpression of RMRP increased NP cell growth and regulated ECM expression through targeting miR‐206.

Taken together, our study showed that RMRP expression was up‐regulated in degenerated NP tissues compared to normal NP samples, and higher RMRP expression was associated with the disc degeneration grade. Ectopic expression of RMRP increased NP cell growth and regulated ECM expression through targeting miR‐206. These results suggested that lncRNA‐RMRP promoted the progression of IDD through targeting miR‐206, providing an attractive new therapeutic approach for the treatment of IDD disease.

## CONFLICT OF INTEREST

There is no conflict of interest statement.
